# Advances in understanding the graft healing mechanism: a review of factors and regulatory pathways

**DOI:** 10.1093/hr/uhae175

**Published:** 2024-06-20

**Authors:** Lixian Wang, Yangmei Liao, Jiming Liu, Tianyun Zhao, Liming Jia, Zhong Chen

**Affiliations:** State Key Laboratory for Efficient Production of Forest Resources, Key Laboratory of Silviculture and Conservation of the Ministry of Education, National Energy R&D Center for Non-food Biomass, Ministry of Education of Engineering Research Centre for Forest and Grassland Carbon Sequestration, College of Forestry, Beijing Forestry University, Beijing 100083, China; State Key Laboratory for Efficient Production of Forest Resources, Key Laboratory of Silviculture and Conservation of the Ministry of Education, National Energy R&D Center for Non-food Biomass, Ministry of Education of Engineering Research Centre for Forest and Grassland Carbon Sequestration, College of Forestry, Beijing Forestry University, Beijing 100083, China; State Key Laboratory for Efficient Production of Forest Resources, Key Laboratory of Silviculture and Conservation of the Ministry of Education, National Energy R&D Center for Non-food Biomass, Ministry of Education of Engineering Research Centre for Forest and Grassland Carbon Sequestration, College of Forestry, Beijing Forestry University, Beijing 100083, China; State Key Laboratory for Efficient Production of Forest Resources, Key Laboratory of Silviculture and Conservation of the Ministry of Education, National Energy R&D Center for Non-food Biomass, Ministry of Education of Engineering Research Centre for Forest and Grassland Carbon Sequestration, College of Forestry, Beijing Forestry University, Beijing 100083, China; State Key Laboratory for Efficient Production of Forest Resources, Key Laboratory of Silviculture and Conservation of the Ministry of Education, National Energy R&D Center for Non-food Biomass, Ministry of Education of Engineering Research Centre for Forest and Grassland Carbon Sequestration, College of Forestry, Beijing Forestry University, Beijing 100083, China; State Key Laboratory for Efficient Production of Forest Resources, Key Laboratory of Silviculture and Conservation of the Ministry of Education, National Energy R&D Center for Non-food Biomass, Ministry of Education of Engineering Research Centre for Forest and Grassland Carbon Sequestration, College of Forestry, Beijing Forestry University, Beijing 100083, China

## Abstract

Grafting is a widely used technique for asexual plant reproduction, especially in agriculture and forestry. This procedure is used to shorten the seedling period, improve the structure of scion branches, and help plants adapt to difficult environments. Although grafting has numerous benefits, several obstacles remain to be overcome. The connection between scion and rootstock is regulated by various factors, including phytohormones and molecular mechanisms, which are crucial for graft healing. This review provides an overview of recent advances in the field of grafting, with a specific focus on the factors and regulatory pathways that influence graft healing. The ultimate goal is to aid understanding of how to achieve successful grafting between plants and create desirable grafting chimeras. We provide an overview of the latest developments in plant grafting, covering aspects related to morphology, physiology, and molecular biology. We also discuss research directions in polyploid breeding and long-distance transfer of small molecules in grafted plants.

## Introduction

Grafting is an ancient agricultural technique that has been used in agricultural production for thousands of years. The Northern Wei Dynasty official Jia Sixie wrote that pear trees grafted onto different rootstocks could produce fruit with varying flavors in the 6th century AD. This was the first recorded instance of grafting used to improve plant characteristics. As grafting technology continued to develop, it began to be used as a routine technology in plant cultivation. There have been many recent reports of the widespread application of grafting in plants such as citrus [1], walnut [2], watermelon [3], and tomato [4]. The results all show that grafting is a very valuable technology.

Grafted seedlings exhibit superior agronomic traits compared to non-grafted seedlings, resulting in higher yields and better fruit quality. Grafting potatoes onto Solanaceae plants has proven to be an effective method for addressing issues related to potato hybridization, including short breeding period, low fruit-setting rate, and sterility [5]. Through reasonable grafting techniques, it is possible to enhance the nutritional value of eggplants [6] and increase the sizes of citrus fruits [7]. Increased yields in grafted crops are often attributed to faster water and nutrient uptake [8] while changing the distribution of nutrients between the nutritional and reproductive organs [9]. In addition, grafting significantly reduces losses by improving resistance to pests and pathogens, conferring tolerance to adverse conditions and stress, and other physiological barriers in grafted plants [10]. Excellent rootstocks can improve the resistance of grafted plants to biotic and abiotic stresses, including but not limited to pests and diseases [11], high salinity [12], low temperatures [13], and flooding [14]. The scion is often selected in high-yielding crops and the rootstock needs to show better adaptability to the environment. Therefore, grafting is a viable solution for the cultivation of high-quality varieties that require meticulous management.

**Figure 1:**
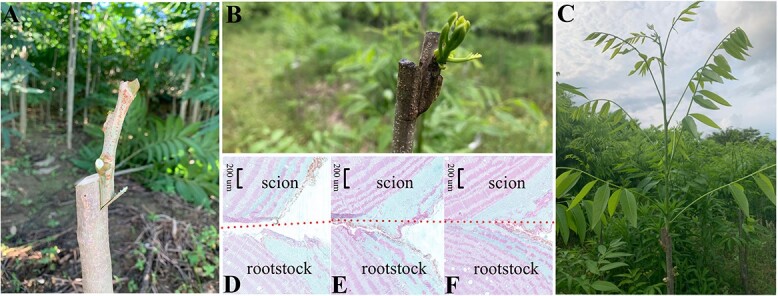
The process of graft healing morphology. External morphological changes and paraffin sections of *Sapindus* graft, initial (**A**, **D**), adherent (**B**, **E**), and totally healed (**C**, **F**). The dotted line indicates the grafted section.

Successful grafting requires the formation of a healthy tissue area between the rootstock and scion systems, allowing the scion to become the new branch system and the rootstock to form the root system (Fig. 1). Numerous studies have determined the details of the graft-healing process through analysis of morphological and metabolic changes in the rootstock and scion at the junction. Hormones, energy substances, and gene families play significant roles in this process. This article provides a comprehensive review of the various stages involved in graft healing, starting from the initial injury response to the reconnection of vascular bundles. The aim is to provide an enhanced understanding of the key physiological processes and molecular mechanisms associated with graft healing.

## Technical elements affecting graft healing

Grafting is a process that involves uniting two parts of a plant, i.e., the root system (rootstock) and the shoot system (scion). The success of grafting is largely dependent on the compatibility between the rootstock and the scion. Incompatible combinations often struggle to develop a well-functioning vascular system [15]. Among a total of 16 grafting combinations involving four species (tomato, pepper, ground cherry, and eggplant), only the interspecific grafting combination of tomato and eggplant has been found to produce healthy grafted plants [16]. Intraspecific grafting is generally thought to have better compatibility than interspecific grafting, and genetic distance is often considered a reliable indicator of grafting compatibility [17]. In agricultural production, the use of interstock (a section of stock between the scion and the rootstock) is a common method to address compatibility issues in interspecific grafting. For instance, Notaguchi *et al.* successfully grafted *Arabidopsis* and tomato by utilizing tobacco as an intermediate rootstock, resulting in the growth of tomato fruits [18]. Recent research has challenged the traditional belief by demonstrating that vascular reconnection can take place between distantly related leguminous plants during the grafting process, while it may fail to occur in some closely related species [19]. These findings indicate that grafting affinity is influenced not only by genetic distance but also by various other factors [20]. For example, Notaguchi *et al.* [18] reported that *Nicotiana* can successfully undergo cross-species grafting with 73 plant species belonging to 38 different families. In addition, they showed that the *NbGH9B3* gene plays a role in promoting graft healing. This discovery offers a novel approach to addressing issues of incompatibility in interspecific grafting. Other biotic factors significantly influence the success of plant grafting by shaping rhizobacterial communities, affecting bacterial diversity, and enhancing network complexity in the rhizosphere [21].

Abiotic factors can influence the success of grafting in plant combinations with relatively high affinity, including external environmental conditions, grafting methods, scion quality, and the expertise of grafting workers [22–24]. Environmental conditions play a crucial role in the survival of grafts [25], and the timing of grafted walnuts significantly impacts plant survival rate and growth [26]. The effects of grafting time on plant growth are primarily mediated through changes in light intensity, temperature, and humidity. The growth of various plants is influenced by varying environmental conditions. It is important to note that different plants have distinct requirements for these variables. The survival of grafts is also influenced by trace elements and nutrient content of the soil. Nitrogen fertilizer significantly impacts stem diameter and dry biomass of grafted cocoa [27] and the absorption of P^+^, K^+^, Mg^2+^, and Ca^2+^ by roots and shoots.

The survival rate of grafts is also influenced by the grafting method, which varies depending on the specific combinations used [28]. Herbaceous plants are often grafted with wedge grafts for better survival [29]. Many woody plants are clonally propagated, and grafting is particularly crucial for woody plants because it significantly shortens the propagation cycle. This has resulted in a greater variety of grafting methods in the production of woody plants. The two primary methods of propagation include bud grafting and branch grafting, with branch grafting proving most effective before bud break and bud grafting being more suitable for plants that have already bud-broken. Various graft types include saddle, wedge, cleft, whip and side grafting methods [30]. It is important to select appropriate grafting methods for different species. For example, saddle grafting is recommended for walnut propagation [22] and wedge grafts are recommended for *Arabidopsis* [29]. In addition, techniques such as micrografting [31, 32] and hypocotyle rootstocks [33] showcase the innovation and precision involved in plant grafting. Successful grafting depends on the adhesion of rootstock and scion cambium tissue. When selecting a grafting method, it is important to consider the effective contact area between the rootstock and scion, nutrient transport, and plant stress resistance [23]. To ensure a high survival rate in production, methods such as grafting immediately after cutting, covering the grafting seedbed with wet wood powder, timely wiping of buds, and maintaining proper water and fertilizer management should be applied.

## Graft healing framework: shifting from wounding responses to vascular connection

The significance of grafting in agriculture and forestry has prompted investigation of the physiological, biochemical, and genetic factors involved in the healing process of grafting. Grafting incompatibility can be categorized into three types: localized incompatibility, translocation incompatibility, and pathogen-mediated incompatibility. Localized incompatibility occurs frequently in the grafting of *Pyrus* and *Prunus*, resulting in a fracture at the grafting interface due to the interruption of cambium and vascular tissue continuity. Translocation incompatibility is characterized by differences in growth speed between the scion and rootstock, which can lead to early arrest of growth and development in grafting. Pathogen-mediated incompatibility is graft failure due to virus and/or phytoplasma presence. The process of compatibility grafting can be divided into four stages: (i) wounding responses and cell wall modifications at the graft interface; (ii) changes in cell division at the graft interface; (iii) the formation and function of plasmodesmata (PD) at the graft interface; and (iv) the vascular connection between the scion and rootstock [34, 35]. In compatible grafting, the mechanism of tissue reunion at the grafted section is influenced not only by the external environment but also by changes in gene expression [20].

### Wounding responses and cell wall modifications at the graft interface

When the scion is grafted onto the rootstock, the joint surface between the scion and rootstock may experience damage to some of the thin-walled cells. This damage leads to protoplasm condensation and the subsequent formation of an isolation layer on the wound surface. The isolation layer is characterized by the presence of brown necrotic tissue. A previous study utilized scanning electron microscopy to perform anatomical investigations on graft-compatible combinations of watermelons and to observe the isolation layer at 11 days after grafting (DAG) [36]. Similarly, the formation of an isolation layer was also found in the early stages of apple grafting [37]. The formation of the isolation layer serves to seal the wound, protecting it against infection by microorganisms. In addition, it helps prevent excessive exudation of organic matter and reduces water evaporation on the grafting surface. The isolation layer is not related to grafting compatibility. The generation of dead cells is a common occurrence in plants after kiwifruit have been damaged by cutting [38].

**Figure 2:**
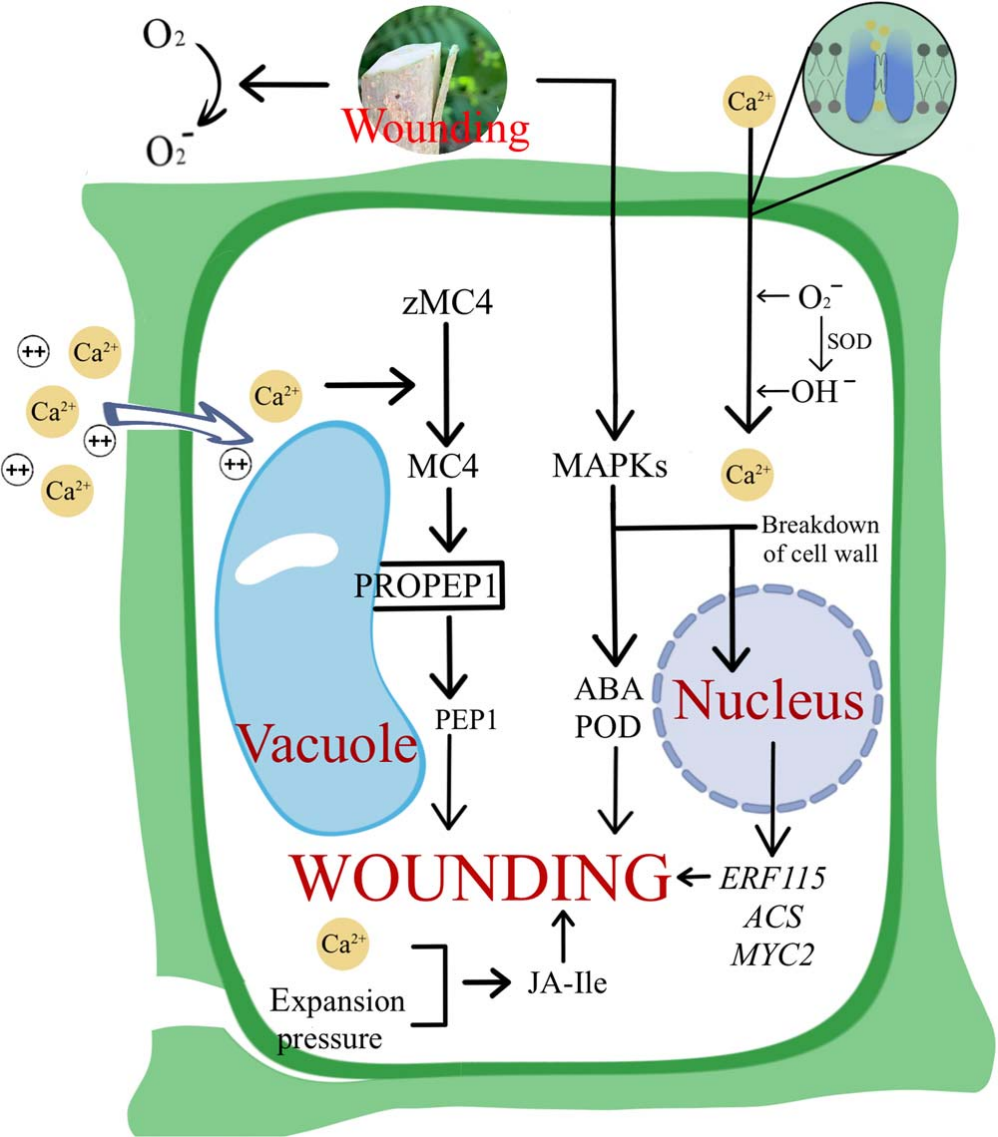
The wounding reaction process in graft healing. Physical damage to grafted plants causes a cascade of defense mechanisms. Intracellular Ca^2+^ and ROS concentrations rise after plant injury, and cell wall modification mechanisms are considerably altered. In the wounding response, phytohormones (jasmonic acid, ethylene) and biological enzymes (SOD, POD) play a crucial role in cell survival.

During the initial phases of grafting, the rootstock and scion undergo changes in structural morphology, which can be observed through the formation of an isolation layer. However, the process of wound recognition and response occurs primarily through intracellular signal transduction (Fig. 2). Changes in the transcriptome due to mechanical injury are found to have significant overlap with those induced by pathogen challenge and abiotic stress [39]. This suggests the existence of a consistent regulatory pathway in the response of plants to wounding. The response to a wound entails modifications in the structure of the cell wall and the identification of wound-related reactions. The initial responses to wounds consist of changes in the levels of reactive oxygen species (ROS) and Ca^2+^, as well as in turgor pressure, cell wall integrity, hormones, and gene expression. ROS operate as second messengers in a variety of biological processes, including programmed cell death, cell cycle, biotic or abiotic stress, and embryogenesis [40]. Furthermore, they play important roles in wound healing. When plant tissue is damaged, the electron transport chain and redox dynamic equilibrium are disrupted, producing a considerable amount of active oxygen ions. The antioxidant system of a plant is activated during this process. The accumulation of ROS leads to changes in the plasma membrane potential, which in turn triggers an influx of Ca^2+^. In *Arabidopsis*, this results in a shift in distribution of the cysteine protease metacaspase 4 (MC4) on the cytosolic side of the vacuolar membrane. The process of transition leads to production of the plant elicitor peptide PEP1, which then binds to extracellular receptors (PEPRs), initiating an immune response [41]. The mitogen-activated protein kinase (MAPK) pathway is a crucial additional mechanism for wound perception. After *Arabidopsis* tissue wounding, the clade-A MAPKs MPK3 and MPK6 are rapidly activated [42], and the MAPK signaling pathway is triggered to participate in intracellular signaling with abscisic acid (ABA) in response to injurious stimuli [43].

After the plant cell wall ruptures, the degradation of pectin and cellulose occurs, resulting in a reduction in cell anisotropic growth [44]. This leads to cell swelling, which alters cell expansion pressure and plasma membrane permeability. Jasmonate-isoleucine (JA-Ile) is a significant regulator of jasmonic acid (JA)-mediated wound responses in plants [45]. It accumulates rapidly after wounding [46]. Changes in cell expansion pressure may be crucial signals for JA-Ile biosynthesis [47]. A recent study showed that treatment of plants with sorbitol or mannitol reduces JA signaling in injured tissues [48, 49], indicating that expansion pressure plays an essential role in JA-Ile biosynthesis. The breakdown products of the cell wall, i.e., oligogalacturonic acid and cellulose dextrin (a glucose polymer), hinder growth and stimulate defense responses [50]. This includes activating the ethylene response factor *ERF115* expression [51], which impacts the regeneration of stem cells in damaged cells. The breakdown of pectin and cellulose alters the overall expression levels of DOF, ANAC, and ERF at the wound site, thus initiating the processes of cell division and differentiation [5, 52].

### Changes in cell division at the graft interface

**Figure 3:**
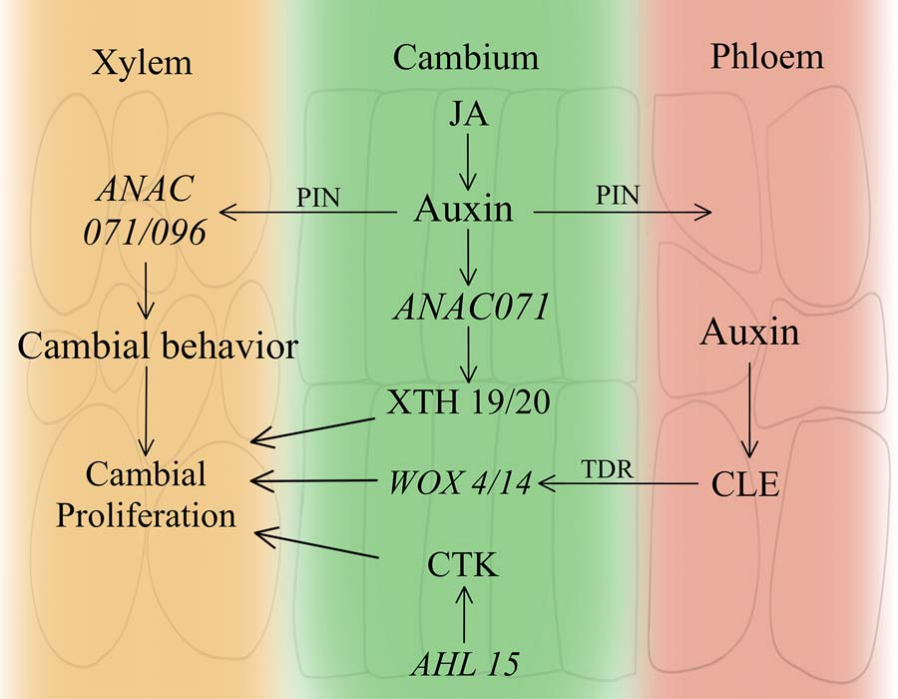
Hormonal-molecular regulatory network of the cell division process. Auxin penetrates secondary xylem cells via active transport, causing them to acquire cambial behavior and begin dividing [53]. The TDIF-PXY signalling module plays an important role in cell division, and it promotes cell proliferation by regulating the expression of the transcription factor WOX4/WOX14. Together with auxin, cytokinins receive regulation from AHL15 and stimulate cell proliferation [54].

Cell division during grafting is regulated by various signals (Fig. 3). When auxin and cytokinin are induced, cells undergo a rapid division phase [55]. At the graft site on the top of the rootstock, parenchymal cells resume division and generate a substantial amount of callus. The scion, on the other hand, initiates division later than the rootstock. Upon receiving stimulation, the cambium produces a significant amount of callus tissue until the rootstock and scion come into contact [56]. In the initial stage of graft healing, callus cells are primarily polygonal, considerably larger in size, and exhibit a high degree of vacuolation. These cells accumulate in a disorganized manner [57].

Plant hormones exert an important influence on the process of cell division in graft healing. After the wound reaction phase, JA is expressed earlier than auxin in injured tissue and activates ethylene response factor109 (*ERF109*) expression, which in turn upregulates the expression of anthranilate synthase α1 (*ASA1*). Zhang *et al.* discovered that in *Arabidopsis* regeneration, increased levels of *ASA1* expression enhance auxin synthesis by regulating the injury signaling pathway [49]. After grafting for 6 hours, PIN proteins (transporters of auxin) in *Arabidopsis* scions exhibit notably high expression in cells surrounding wounds [58], and auxin is transported via this protein into secondary xylem cells at the site of injury. This facilitates the acquisition of cambium-like behavior and initiates proliferation in these cells. The spatial pattern of cambium marker genes around *Arabidopsis* wound sites is similar to that of auxin-responsive genes [58, 59]. This suggests a close relation between cambium formation and auxin [35]. Pitaksaringkarn [60] conducted an experiment where *Arabidopsis* inflorescence stems were incised and treated with IAA or distilled water. The results showed that the expression of Pro*ANAC071*::*GUS* was higher in the IAA treatment group, indicating that the accumulation of auxin stimulates the production of *ANAC071*. *ANAC071*, a transcription factor from the NAC family, directly induces the expression of the xyloglucan endotransglucosylase/hydrolase family members XTH19 and XTH20, which catalyze the molecular grafting and/or hydrolysis of xyloglucans in cell walls [60]. Using pTDR/PXY::*GUS* as a cambium marker, Matsuoka observed the development of cambial cells in partially incised stems. The findings indicate that *ANAC071* and *ANAC096* can additionally stimulate cell proliferation, causing secondary xylem cells to change into cambium cells [53]. Clavata3/embryo surrounding region-associated (CLE) peptide signaling, a tracheal element differentiation inhibitory factor (TDIF), maintains cambium cell proliferative activity during embryonic growth. Phloem-derived CLE41 and CLE44 bind to TDIF receptors (TDR/PXY), localize to the cell membrane of cambium cells, and promote cell proliferation [61, 62]. In cell division, *WOX4*, *WOX13*, and *WOX14* of the WUSCHEL-related homeobox (WOX) family are involved through distinct pathways. Ikeuchi *et al.* [63] investigated the function of *WOX13* in promoting tissue regeneration and organ reattachment in *Arabidopsis*. The study employed a reporter transactivation assay to examine the activity of the *WOX13* promoter when *WIND1* was overexpressed in *Arabidopsis* culture cells. The results show that *WIND1* induces *WOX13*, which in turn upregulates *WIND2* and *WIND3*, promoting cell reprogramming. On the other hand, *WOX4* and *WOX14* play similar roles in cell proliferation downstream of the auxin-PXY pathway [64].

Cytokinins (CTKs) have a crucial positive impact on graft healing. Recent research has discovered a novel pathway that stimulates cambium development in *Arabidopsis*, in which AT-hook motif containing nuclear localized 15 (*AHL15*) plays a vital role in enhancing cell division. That study utilized the cytokinin (CTK) in *Arabidopsis* stem segments response reporter to determine the response of cytokinin to *AHL15* and reported that the cytokinin synthesis genes (*LOG4*, *LOG5, IPT3*, and *IPT7*) were notably downregulated in *ahl15* mutant, leading to a decrease in cambium cell division activity [54]. These findings suggest that AHL15 acts as an upstream regulator of cytokinin synthesis. In *Arabidopsis* stem segments, Iwase *et al.* discovered that overexpression of *WIND1* is capable of inducing unorganized cell proliferation and maintaining the dedifferentiated state. Through the use of a synthetic reporter, the two-component-output sensor (TCS):GFP, it was confirmed that the B-type ARR-mediated cytokinin response increased within 24 hours in pericycle and surrounding cells near the wound site. Upon wounding, the activation of *WIND1* and its functional homologues triggers a localized cytokinin response, ultimately leading to cellular dedifferentiation [65]. Various studies have highlighted the crucial role of cytokinins in cell division and the formation of healing tissue. In *Arabidopsis*, cytokinin signalling stimulates histidine kinase receptors and initiates a phosphorelay transduction process [66, 67], which in turn activates type-B *Arabidopsis* response regulators (ARRs) [68–70]. Type-B ARR transcription factors are capable of increasing auxin levels by directly regulating the expression of *YUCs* [71]. These transcription factors are vital for cell division and callus formation, further aiding the healing process. Liu *et al.* utilized time-lapse imaging to illustrate that the proper functioning of auxin and cytokinin in *Arabidopsis* is dependent on auxin for the co-regulation of cell division and differentiation [72]. This co-regulation is believed to be associated with the expression of d-type cell cycle proteins, which impacts the cell cycle of cambium stem cells [73, 74]. However, the precise mechanism by which cytokinins and auxins interact in this process remains unclear.

### Formation and permeability regulation of plasmodesmata

**Figure 4:**
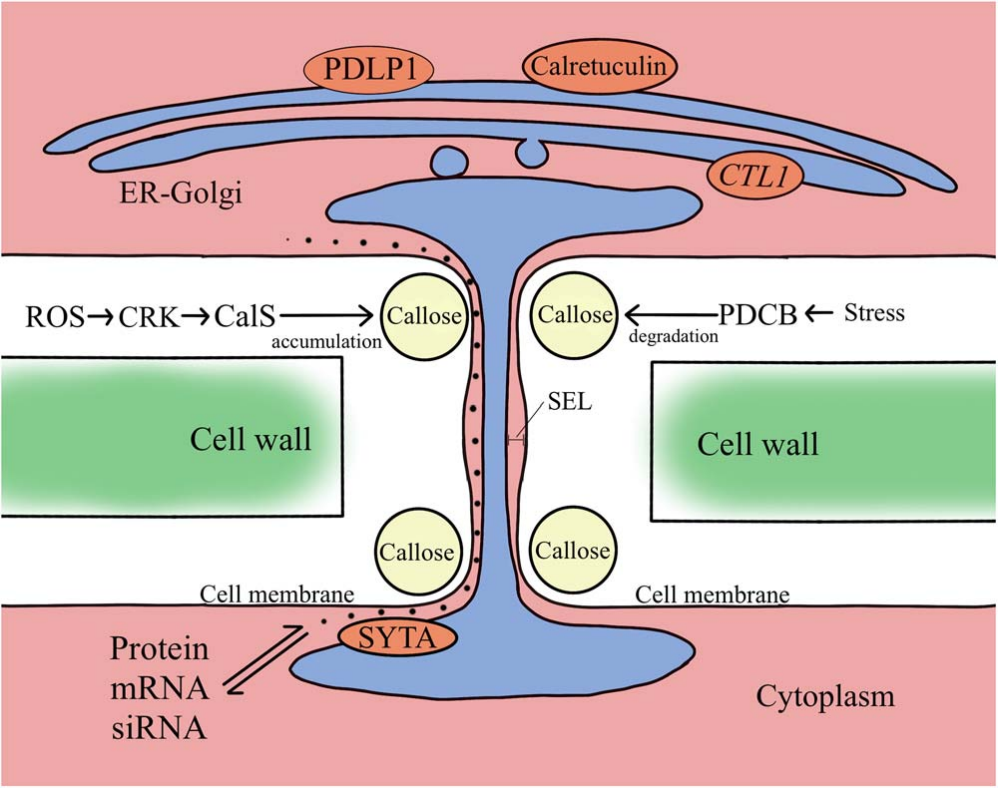
The formation and permeability regulation of plasmodesmata. The formation and opening of dynamic PD is controlled by intracellular components such as PM-lipid rafts, ER-bristle tubes, ER-Golgi apparatus, ER-PM contacts, and cell walls. Plasmodesmata serve as conduits for horizontal gene transfer (HGT) between rootstock and scion [75].

Symplasm structural domains play crucial roles in facilitating cellular communication between proliferating cells in the transplanted area and are essential for appropriate tissue development (Fig. 4). Intercellular homogenous connections, also known as PD, are formed by plant cells between adjacent cells to aid in the transfer of large molecules, such as proteins, mRNA, and siRNA, as well as low-molecular-weight nutrients [75]. Upon contact with the plasma membrane, the endoplasmic reticulum (ER) induces relaxation and thinning of the cell wall. This results in protrusion of the plasma membrane and ER to form tubules, which eventually penetrate the cell wall and fuse with the plasma membrane of neighboring cells, thus forming PD. The PD plays important roles in coordinating the various developmental stages of plant growth.

The ER-Golgi pathway is essential for the formation of PD. Inhibition of the ER-Golgi secretion pathway results in the retention of PD-localized proteins in the ER, which eliminates the localization mode of PDLP1 [76]. This suggests that the ER-Golgi secretion pathway is responsible for providing a mechanism for PD positioning information. This is supported by a number of previous studies. One such study found that calreticulin in the ER can modify the structure of the ER to target PD biosynthesis [77]. Key genes involved in the biosynthesis of PD have been discovered. One such gene is choline transporter-like gene 1 (*CTL1*), which has been identified as a regulatory gene involved in the process of PD biogenesis. *CTL1* is found in the trans-Golgi network (TGN), and its deficiency causes the mislocalization of PD to intracellular TGN compartments. This in turn impairs the morphology of PD, leading to disruption of secondary PD biogenesis [78]. Typically, only the overall visible effects of PD can be studied. The process of biogenesis and functional regulation of individual PDs is of great importance, but the nanometer scale of individual PDs is far beyond the light diffraction limit, so it is difficult to resolve this characteristic experimentally [79].

The formation of symplasm domains is crucial for successful grafting. The symplasm transport efficiency of PD is significantly enhanced in compatible combinations compared to incompatible grafts [80]. According to the classical model, the PD pore size exclusion limit (SEL) is determined by the gap between the ER membrane and the cell membrane. This means that type I PD, which have a smaller gap, are less permeable than type II PD with a larger gap [81]. However, recent studies have challenged this model. Electron tomography experiments have shown that type I PD at the interface of the rhizosphere endocortex of *Arabidopsis* has a higher capacity for symplastic unloading [82]. This suggests that the permeability of the PD is regulated by a more complex mechanism than previously thought [83].

**Figure 5:**
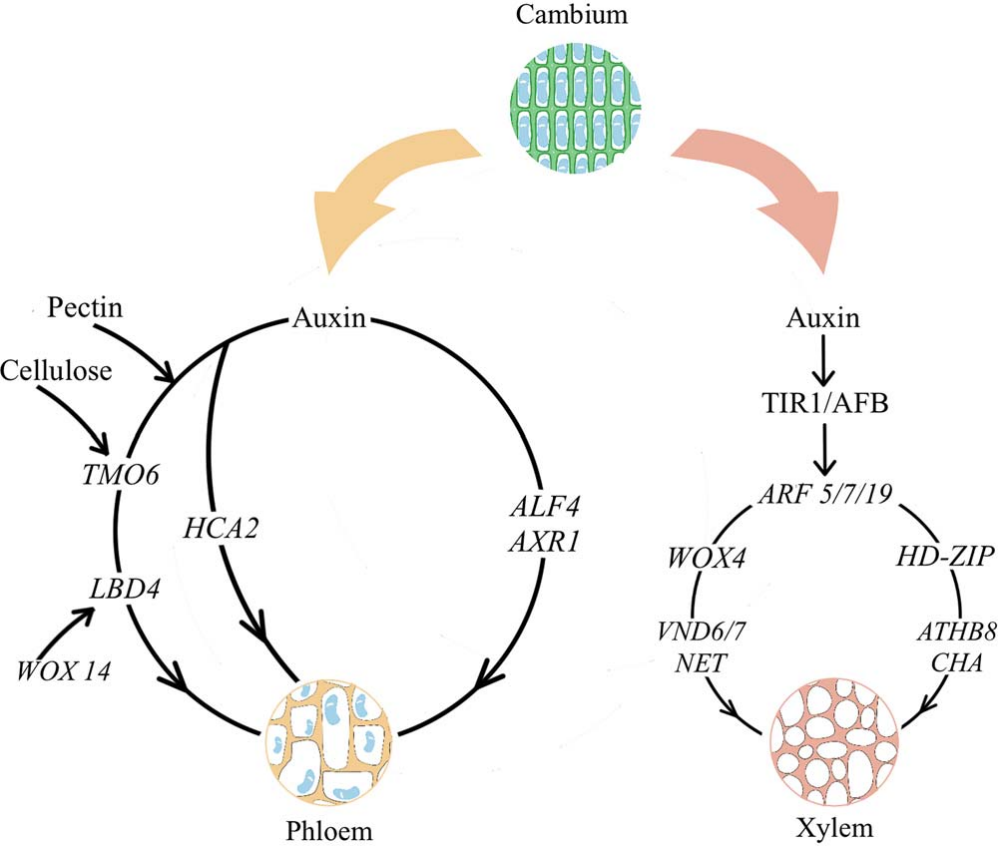
Regulation network of vascular reconnection in grafted plants. Both xylem and phloem differentiation were affected by auxin. The expression of LBD4, HCA2, and ALF4 promoted the transformation of cambial cells into phloem cells. Additionally, WOX4 and HD-ZIP III played crucial roles as regulators in xylem differentiation.

Previous studies have demonstrated the involvement of callose in the regulation of the permeability of PD. The accumulation of callose is precisely regulated by callose synthases (CalSs), specific PD-localized β-1,3-glucan synthase (PDBGs), and PD callose binding proteins (PDCBs) [84–87]. Plants possess multiple CalSs, which regulate callose biosynthesis in distinct tissues and respond to both biotic and abiotic stress processes. The Cys-rich receptor-like kinase (CRK) is a significant mediator of intercellular communication and extracellular environmental information exchange. CRK2 phosphorylates CalS1, thereby promoting callose accumulation [88]. In addition, CRK plays a role in regulating PD permeability by responding to the ROS-mediated emergency signaling pathway [89]. Four genes (i.e., *AtBG_pap*, *PDBG1*, *PDBG2*, and *PDBG3*) are closely related to the degradation of callose [90, 91]. In *Arabidopsis*, an excess of iron and copper ions can significantly alter the concentrations of CalSs and PDBGs, ultimately affecting the efficiency of intercellular information transmission [92, 93]. This adaptation allows the plant to respond more quickly to environmental stressors.

Unlike callose, certain proteins can modify the permeability of PD by altering its structure. Synaptotagmin A (SYTA) from the SYT1 family has been studied most extensively among these proteins. Mutations in *syta* lead to changes in the three-dimensional configuration of the ER, ultimately blocking PD permeability [94]. Another protein, RTNLB, plays a crucial role in regulating the opening and closing of PD by facilitating ER contraction and creating a favorable lipid environment for PD formation [95, 96]. A recent study demonstrated that mitochondrial proteins, which are linked to targets of rapamycin (TOR) metabolic signaling, can also impact permeability [97]. PD dysfunction is likely responsible for the low survival rate of interspecific grafts [98]. In successful grafting, PDs serve as conduits for horizontal gene transfer (HGT) between the rootstock and scion [99, 100]. Grafting presents an opportunity for the creation of allopolyploid species and novel combinations of organelle genomes.

### Reconnection of vascular tissue between rootstock and scion

Once the communication network between the rootstock and the scion is established, the original cambium of the rootstock undergoes differentiation (Fig. 5). Inward differentiation leads to the formation of numerous xylem cells and the appearance of vessels, while outward differentiation results in the formation of phloem and the appearance of sieve tubes. The reconnection of vascular bundles enables long-distance material transport and signal transmission between rootstock and scion, facilitating normal growth of the grafted plant. Carboxy-fluorescein diacetate (CFDA) is commonly used to identify the phloem reconnection process, while acid fuchsin is used to identify the xylem reconnection process [101, 102]. It is important to note that phloem reconnection often occurs earlier than xylem reconnection and is regulated by distinct hormone–molecule interaction networks.

**Figure 6:**
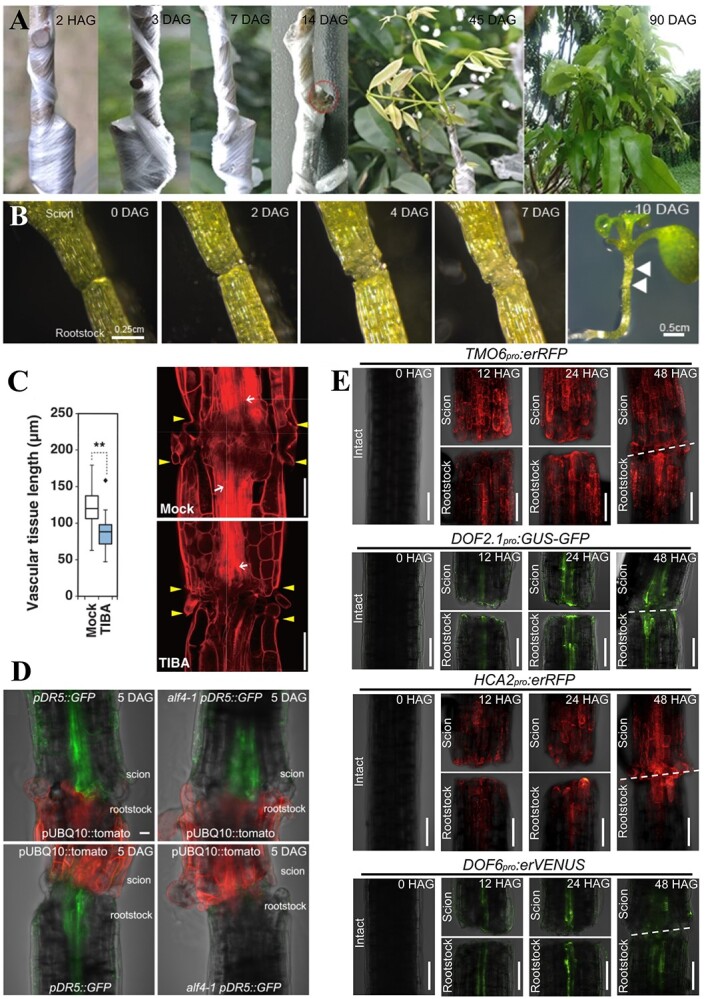
Morphological changes during graft union development. **A** Morphological changes of the litchi-compatible graft at 2 h, 3 d, 7 d, 14 d, 45 d, and 90 d after grafting [103]. **B** The *Arabidopsis* graft junction monitored from 0 to 7 days after grafting (DAG) using brightfield optics [57]. **C** Box plots indicate vascular tissue length between the different *Arabidopsis* treatments. Longitudinal optical sections across the vascular tissue of the mPS-PI-stained graft union zone at 7 DAG treated with the indicated inhibitors or phytohormone. Mock, 0.1% (v/v) dimethylsulfoxide; TIBA, 100 μM. Yellow arrowheads and white arrows indicate the positions of the cut surface and the differentiated xylem vessel, respectively [104]. **D**  *ALF4* is required in the rootstock to activate vascular auxin response in *Arabidopsis*. Col-0 or *alf4* scions in the *pDR5rev::GFP-ER* background were grafted to *pUBQ10::PM-tdTomato* rootstocks, or grafted reciprocally [57]. Scale bar, 25 μm. **E**  *TMO6pro:erRFP, HCA2pro:erRFP, DOF2.1pro:GUS-GFP,* and *DOF6pro:erVENUS* are upregulated 12–48 h after *Arabidopsis* grafting. The gap or dashed line indicates the graft junction. Scale bar, 100 mm [5].

After a series of tissue-specific cellular events, vascular tissue differentiates and reconnects damaged vasculature to restore systemic transport systems (Fig. 6). According to the channelization hypothesis, auxin is involved in polarizing cell differentiation through directed intercellular flow. The auxin transport inhibitor TIBA (2,3,5-triiodobenzoic acid) significantly inhibits the healing process of grafted plants [104]. Taken together, these findings show that auxin is essential for vascular reconnection. Another *Arabidopsis* study reported that the process of phloem reconnection is not significantly influenced by cytokinins and ethylene in rootstocks [57]. However, a reduced response to auxin significantly impacts phloem reconnection. In that study, the authors grafted wild-type scions expressing pSUC2::GFP onto rootstocks of 45 genotypes and examined the timing of fluorescence in the rootstocks to determine whether phloem reconnection was affected. Four auxin-responsive mutants (*alf4*, *axr1*, *iaa18*, and *tir1-afb2-afb3* triple mutants) showed a strong impact on phloem reconnection. The expression of aberrant lateral root formation 4 (*ALF4*) and auxin resistant 1 (*AXR1*) is necessary for rootstocks, but the absence of these genes in mutant scions (*alf4* and *axr1*) does not impact phloem reconnection. To date, key genes showing specific expression in scions and significantly impacting phloem reconnection have not been identified. The promotion of phloem connections via cell wall modification is another mechanism in which auxin plays a role. This is achieved through pectinolysis, leading to the upregulation of transcription factors for DOFs at the wound site and surrounding cells. This process is inhibited in the absence of auxin [41]. DOF transcription factors responsible for cell wall and vascular remodeling, including *HCA2* [58, 105] and *TMO6* [106], are rapidly activated after grafting. *HCA2* promotes phloem cell formation, while *TMO6* and *WOX14* coregulate *LBD4* expression to define phloem boundaries [107]. Compared to other auxins, auxin has a more profound effect on phloem development [107].

Auxin also plays a key role in xylem reconnection. Intracellular and intercellular auxin concentrations have opposite effects on xylem development [108]. That study used auxin mobile carrier mutations and reported that an increase in intercellular auxin concentration inhibits xylem differentiation, while an increase in intracellular auxin concentration has a promoting effect. Intercellular auxin concentration forms a cell-surface complex through TMK proteins and ABP1, which couples intercellular signaling to cytoplasmic signaling [109]. Intracellular auxin in *Arabidopsis* promotes graft healing by binding to the transport inhibitor response 1/auxin signaling F-box (TIR1/AFB) receptor [110]. TIR1/AFB proteins act as negative regulators of various auxin response factors (ARFs), leading to increased ARF activity and increased intracellular auxin concentration [111]. A previous study reported consistent and strong expression of *ARF5/7/19* in the vascular cambium of *Arabidopsis*. However, the downregulation of gene subsets encoding *WOX4* and *HD-ZIP III* transcription factors was observed, along with a reduction in secondary vascular cells in triple mutant plants [112]. *WOX4* is a key regulator of xylem reconnection in tomato and pepper xenografting experiments; it regulates both *VND6/7* and *NET* expression, which are responsible for xylem differentiation. In addition, *wox4* mutants exhibit noticeable xylem defects [15]. The HD-ZIP III family of transcription factors plays essential roles in xylem differentiation. Inhibiting the expression of five specific HD-ZIP III transcription factors (*ATHB8*, *CHA*, *PHB*, *PHV*, *REV*) significantly hinders xylem differentiation [112, 113]. The process of xylem differentiation is promoted by three positive-feedback loops of Auxin-ARF-HD-ZIPIII [114, 115].

## Roles of sugar and asymmetric expression in graft healing

The presence of sugar is a crucial factor in the process of graft healing. Herbaceous plants are often grafted with wedge grafts. Studies in *Arabidopsis* have shown that grafting medium containing 0.5% sucrose more effectively promotes graft healing and survival than medium containing 0% or 1% sucrose [116]. In the heterograft of cucumber onto pumpkin, compared with grafted etiolated tissue without sugar, the xylem connection was achieved 1 day earlier by the addition of 0.5% glucose, whereas there was no significant change in the normal tissue with exogenous sugars compared with the normal tissue without sugar treatment. [102]. Research on *Rhodiola rosea* shows higher sucrose levels (20 g/l) promote callogenesis in golden root plants, enhancing callus formation. Optimal sucrose concentration is crucial for plant graft healing [117]. This suggests that an appropriate sugar concentration is necessary for successful plant graft healing. Sugars play crucial roles as respiratory substrates in energy production and the synthesis of biological macromolecules [118]. In the heterograft of cucumber onto pumpkin, sugars promote graft healing through the TOR pathway [102], with a significant role of Glc-TOR in the grafting process. Grafting experiments with *Nicotiana* and *Arabidopsis* showed that the subclade of β-1,4-glucanase secreted extracellularly plays a crucial role in cell wall remodeling at the grafting interface. Surprisingly, overexpression of β-1,4-glucanase contributed to the successful grafting of *Nicotiana* and tomato to obtain tomato fruits on *Nicotiana* rootstock [18]. This enzyme also plays a significant role in targeting cellulose for the survival of *Arabidopsis* interfamily grafts, and grafting studies in *Vitis* have shown that cellulose has a crucial role in the healing process [119]. In *Arabidopsis* stem segments, cellulase also increases TMO6 activity independently of auxin, indicating the importance of polysaccharide-dominated cell wall remodeling for the successful healing of grafts [120].

Physical cutting results in cellular asymmetry above and below the cut site, leading to distinct physiological responses in scion and rootstock cells that influence graft survival. The expression of genes promoting graft healing is observed earlier in scion cells, while *Arabidopsis* rootstock cells show a 24 h delay in their activation [58]. Successful graft combinations can significantly improve the levels of asymmetric gene expression, thus paving the way for further research on graft healing. This may be linked to changes in auxin concentration [121], which can alter the expression of certain genes in both the scion and rootstock and lead to varying callus activities. Transcriptomics analyses have confirmed the significant influence of the asymmetric response of JA [122] and sugar [102] at the graft site on the healing process. Although there is still debate regarding the role of this asymmetric response in healing, its crucial significance in the process of grafting is undeniable. A study that used fluorescence labeling technology found that 83.3% of new cells in the callus originated from the scion [123]. Another study compared gene expression in grafted and non-grafted *Arabidopsis* stem segments after cutting and observed significant differences in gene expression below the cut position. Specifically, only rootstocks showed phloem reconnection promoted by *ALF4* and *AXR1* [57], while grafted plants exhibited increased expression of *TMO6* and *HCA2*, which are associated with phloem development, 6 h after excision [58]. Although there have been some reports regarding asymmetry, the process by which it is established is still unclear. Further studies are required to understand the formation of this response, including the signals conveyed by the rootstock and scion during the initial grafting period and how these signals are detected and promote healing. Further research addressing these issues is crucial.

**Table 1:** Functional summary of genes involved in graft healing

**Species**	**Name**	**Gene number**	**Gene function**	**References**
*Arabidopsis thaliana*	*AHL15*	AT3G55560	Enhance cell division.	[54]
	*ALF4*	AT5G11030	Promote phloem reconnection in rootstocks.	[57]
	*ANAC071*	AT4G17980	Promote cell division; involved in the process of ‘cambialization’.	[53, 60]
	*ANAC096*	AT5G46590	Involved in the process of ‘cambialization’.	[53]
	*ARF5/7/19*	AT1G19850 AT5G20730 AT1G19220	Play an important role in cell division and xylem differentiation.	[112]
	*ASA1*	AT5G05730	Enhance auxin synthesis.	[49]
	*AXR1*	AT1G05180	Promote phloem reconnection in rootstocks.	[57]
	*CalS*	AT1G05570	Regulate callose biosynthesis.	[88]
	*CTL1*	Dmel_CG1311	Provide cellular desmosis location information.	[78]
	*ERF109*	AT4G34410	Receive and conduct JA signals.	[49]
	*ERF115*	AT5G07310	Involved in cell wall modifications.	[51]
	*HCA2*	AT5G62940	Involved in cell wall remodeling and vascular remodeling.	[58]
	*ATHB8*	AT4G32880	Regulate xylem differentiation.	[112]
	*IPT3/7*	AT3G63110 AT3G23630	Promote cytokinin synthesis.	[54]
	*LBD4*	AT1G31320	Define phloem boundaries.	[107]
	*LOG4/5*	AT3G53450 AT4G35190	Promote cytokinin synthesis.	[54]
	*TMO6*	AT5G60200	Promote phloem cell formation.	[106]
	*WIND1*	AT1G78080	Lead to cellular dedifferentiation.	[65]
	*WOX13*	AT4G35550	Promote cellular reprogramming and organ regeneration; orchestrate the transcriptional induction of cell wall-modifying enzyme genes.	[63]
	*WOX4/14*	AT1G46480 AT1G20700	Promote cell division.	[64]
*Solanum lycopersicum*	*SlWOX4*	LOC100301933	Regulate xylem differentiation.	[15]

## Perspectives

Grafting is essential for boosting plant resilience against both biotic and abiotic stresses through the stimulation of physiological and molecular alterations. It can improve a plant’s ability to fend off diseases and pathogens by facilitating genetic resistance to soilborne pathogens, enlisting microbial antagonists, and releasing antifungal compounds in the rhizosphere. This, in turn, diminishes the reliance on synthetic fungicides and encourages sustainable agricultural methods. The interaction between rootstock and scion in grafted plants can result in the transfer of multiple resistant genes, bolstering the plant’s defense mechanisms against a range of stresses, including biotic factors [31]. Grafting also impacts gene expression linked to stress tolerance, particularly in shoot apical meristem cells, leading to enhanced drought tolerance due to altered gene expression patterns. Research on grapevine, cucumber, tomato, and cucumber-cucumber grafting systems demonstrates that grafted plants display increased stress tolerance by improving water content, photosynthesis, antioxidant defense, and hormonal regulation [124–127]. Grafting plays a crucial role in enhancing biotic and abiotic tolerance by regulating the morphology of roots, stems, and leaves, reducing transpiration through modulation of stomatal closure, activating osmoregulation, enhancing antioxidant systems, and altering gene expression and phytohormone levels [128, 129].

Grafting propagation is a useful technique for maintaining the desirable characteristics of parent plants. It offers a high reproduction coefficient and fast reproduction speed, making it an important tool for improving crop varieties. Through grafting, rootstocks can introduce resistance to pathogens, improve plant vigor, and enhance stress tolerance, ultimately increasing crop quality, yield, and resilience to environmental challenges. Furthermore, it can lead to the transfer of entire plastid, mitochondrial, or nuclear genomes between species, resulting in new combinations of nuclear and organellar genomes, or even the creation of new species with allopolyploidy [75]. Therefore, it is an unconventional but noteworthy breeding method. While there is a wide range of research on grafting, the ability of tissues to heal successfully is a crucial aspect of all studies. Previous research has identified specific hormonal responses and gene expression patterns corresponding to the morphological changes that occur during graft healing (Table 1). During the process of graft healing, stress signals are generated due to cell damage and changes in mechanical pressure at the interface. These signals play a crucial role in initiating cell regeneration. In addition to the cambium, callus can also be produced by cortical parenchymal cells, secondary xylem cells, and secondary phloem cells. As callus increases, the rootstock and scion cells come into contact and exchange information through PD. This process is regulated by PD permeability and may lead to horizontal gene transfer. Once an intercellular communication network is established, the cells differentiate into vascular tissue. The reconnection of vascular bundles between the rootstock and scion signifies the completion of the graft-healing process. Although the processes are not strictly separated, the above-mentioned phases encompass the basic biological events.

Outstanding problems in plant graft healing include understanding the mechanisms of graft incompatibility, nutrient loss post-grafting, especially in heterografts, and the need to optimize healing conditions for grafted plants, such as light exclusion and temperature regulation. Additionally, the molecular mechanisms underlying successful healing after heterografting remain largely unknown. These challenges highlight the complexity of grafting processes, including wound repair, hormonal signaling, and vascular regeneration within the graft union zone. Addressing these issues will enhance the efficiency and success rates of plant graft healing techniques.

The biological framework of graft healing is well known, but there are still many unknowns in the healing process. First, plants exhibit gene expression differences in physically cut tissues during the early stages of grafting, and the cause of this phenomenon is still unknown. Second, the callus formed by grafting differs in terms of physiological and molecular properties from the callus formed by the plant in culture *in vitro*. The reasons for these differences have yet to be determined. Finally, in many species, such as *Arabidopsis* [57], Watermelon [36], etc, the phloem often connects earlier than the xylem during the process of vascular bundle reconnection. The impact of phloem reconnection on xylem reconnection is still unclear. Further detailed studies are required to determine the mechanism of graft healing. Combined with single-cell sequencing technology, these questions will likely be answered in the near future. Applications of single-cell sequencing technology in plant graft healing mechanisms involve accurately identifying graft-mobile transcripts, understanding tissue reunion processes through scRNA-sequencing data [130], and characterizing heterogeneity in cellular systems using scRNA-seq [98]. To gain key insights into plant grafting success with scRNA-seq, experimental design should focus on accurately identifying transcripts by considering factors such as read-depth variability, error rates, and multiple single-nucleotide polymorphisms (SNPs) per transcript [130]. Additionally, sequencing samples should include samples before and after vascular reattachment, which is crucial for graft healing and molecular transport across graft site junctions. scRNA-seq contributes to understanding the molecular mechanisms underlying successful grafting in plants through processes such as cell isolation, cell type annotation, and analysis of cell function [131].

In grafted plants, there is an exchange of genetic material and information transfer between the scion and the rootstock. Various substances, such as water, sugars, mineral nutrients, proteins, RNA, and other macromolecules that determine biological traits, can travel long distances across graft unions. Many mRNAs are transported through the phloem, influencing organ growth and development across considerable distances [5, 106, 132]. Initially, the regulation of endogenous transcript migration was thought to be governed by specific motifs, such as PTB motifs [133] and TLS motifs [106]. However, a study reported experimental results contradicting this suggestion and demonstrating that cytosine methylation indeed plays a significant role in mRNA transport [134]. Furthermore, it is important to note that mRNA movement relies on the formation of a ribonucleoprotein complex (RNP) between mRNA and RNA-binding proteins. This complex serves as the foundation for the transportation of mRNA [135]. This perspective is supported by experiments that showed that the PbCCT complex can assemble PbPTB3 and PbWoxT1 to create an RNP complex in the phloem tissue of *Pyrus betulaefolia*, which aids the intercellular transport and phloem growth of the PbWoxT1-PbPTB3 RNP complex over long distances [136]. The mechanisms underlying the endogenous factors that initiate mRNA transport between rootstock and scion remain incompletely understood.

In addition to mRNA, microRNAs (miRNAs) and short interfering RNAs (siRNAs) are also transported over long distances between rootstock and scion through the phloem [134]. Noncoding RNA transport is an important mechanism for mediating epigenetic effects alongside DNA methylation [137]. Tomato grafting studies have reported siRNA-mediated epigenetic effects. Experiments have demonstrated that the effect of *msh1* (mutant rootstock) on tomato-grafted plants can be inherited for more than five generations, indicating the potential of epigenetics in plant breeding [138]. There is significant interest and excitement within the scientific community regarding epigenetics. However, the application of epigenetics to crop breeding is a relatively new field due to the inherent instability of epigenetic changes [139, 140]. In future research, grafting may play a crucial role in exploring the reprogramming of gene expression.

Grafting has been identified as a plausible method for asexual speciation. Organellar genomes experience short-distance transfer and recombination at graft junctions [100, 141]. Due to the maternal inheritance in most species, plastids and mitochondria cannot be separated by hybridization. Therefore, the only viable method to acquire new combinations of plastids and mitochondria is through protoplast fusion [75]. Grafting is a highly effective method for combining parental plastids and nonrecombinant mitochondria, and it should be noted that this process differs significantly from protoplast fusion [142]. A study that investigated the possibility of nuclear DNA transfer during grafting demonstrated that new allopolyploid cells are formed at the graft junction. In that study, plants grown from these polyploid cells were able to produce fertile offspring, thus highlighting the significant role of grafting in plant breeding [99].

CRISPR-Cas9 technology offers significant potential in plant grafting by allowing precise gene editing without the need for double-strand breaks or donor repair templates [143]. This innovative tool enables targeted modifications in plant genomes, leading to the development of desired traits like disease resistance, improved yield, and enhanced quality. By facilitating precise gene editing in various crop species, CRISPR-Cas9 technology has the potential to enhance plant grafting, potentially improving graft compatibility and overall plant health [144]. Gene editing through grafting provides an advantage by eliminating the need for multiple generations to remove transgenes or regenerate plants from transfected protoplasts [145]. Recent research demonstrating successful grafting in monocotyledonous species [146] suggests that this technique, utilizing mobile Cas9-TLS and gRNA-TLS fusions, could be applied to major crops such as maize, wheat, and rice.

## Data Availability

The datasets presented in this study can be found in online repositories. The names of the repository/repositories and accession number(s) can be found in the article.
